# Cloning, expression and characterization of an aryl-alcohol dehydrogenase from the white-rot fungus *Phanerochaete chrysosporium* strain BKM-F-1767

**DOI:** 10.1186/1471-2180-12-126

**Published:** 2012-06-28

**Authors:** Dong-Dong Yang, Jean Marie François, Gustavo M de Billerbeck

**Affiliations:** 1 Université de Toulouse; INSA, UPS, INP; LISBP, 135 Avenue de Rangueil, Toulouse, F-31077, France; 2 INRA, UMR792 Ingénierie des Systèmes Biologiques et des Procédés, Toulouse, F-31400, France; 3 CNRS, UMR5504, Toulouse, F-31400, France; 4 INP-ENSAT, Avenue de l’Agrobiopole, Castanet-Tolosan Cedex, F-31326, France

**Keywords:** *AAD*, Aryl-alcohol dehydrogenase, Lignocellulosic hydrolysates, Lignin, Flavours, Fragrances, *Phanerochaete chrysosporium*

## Abstract

**Background:**

The white-rot fungus *Phanerochaete chrysosporium* is among the small group of fungi that can degrade lignin to carbon dioxide while leaving the crystalline cellulose untouched. The efficient lignin oxidation system of this fungus requires cyclic redox reactions involving the reduction of aryl-aldehydes to the corresponding alcohols by aryl-alcohol dehydrogenase. However, the biochemical properties of this enzyme have not been extensively studied. These are of most interest for the design of metabolic engineering/synthetic biology strategies in the field of biotechnological applications of this enzyme.

**Results:**

We report here the cloning of an aryl-alcohol dehydrogenase cDNA from the white-rot fungus *Phanerochaete chrysosporium*, its expression in *Escherichia coli* and the biochemical characterization of the encoded GST and His_6_ tagged protein. The purified recombinant enzyme showed optimal activity at 37°C and at pH 6.4 for the reduction of aryl- and linear aldehydes with NADPH as coenzyme. NADH could also be the electron donor, while having a higher *Km* (220 μM) compared to that of NADPH (39 μM). The purified recombinant enzyme was found to be active in the reduction of more than 20 different aryl- and linear aldehydes showing highest specificity for mono- and dimethoxylated Benzaldehyde at positions 3, 4, 3,4 and 3,5. The enzyme was also capable of oxidizing aryl-alcohols with NADP ^+^ at 30°C and an optimum pH of 10.3 but with 15 to 100-fold lower catalytic efficiency than for the reduction reaction.

**Conclusions:**

In this work, we have characterized the biochemical properties of an aryl-alcohol dehydrogenase from the white-rot fungus *Phanerochaete chrysosporium*. We show that this enzyme functions in the reductive sense under physiological conditions and that it displays relatively large substrate specificity with highest activity towards the natural compound Veratraldehyde.

## Background

Lignin is, after cellulose, the second most abundant terrestrial biopolymer, accounting for approximately 30% of the organic carbon in the biosphere [1]. The biodegradation of lignin plays a crucial role in the earth’s carbon cycle. Unlike cellulose and hemicellulose, this amorphous and insoluble aromatic material lacks stereoregularity and is not susceptible to hydrolytic attack. In nature, the white-rot fungus *Phanerochaete chrysosporium* is among the small group of fungi that can completely degrade lignin to carbon dioxide while leaving the crystalline cellulose untouched [2].

Lignin degradation by *P. chrysosporium* is initiated by an array of extracellular oxidases and peroxidases, such as the multiple isoenzymes of lignin peroxidase (LiP) and manganese-dependent peroxidase (MnP) [3-6]. Both LiP and MnP require extracellular H_2_O_2_ for their catalytic activity. One likely source of H_2_O_2_ is the copper radical oxidases, such as glyoxal oxidase [7-9]. In addition to the copper radical oxidases, the FAD-dependent extracellular aryl-alcohol oxidases (Aaop) catalyze the oxidation of aryl-alcohol derivatives into their corresponding aldehydes with the concomitant reduction of O_2_ to H_2_O_2_[6,10]. The Aaop substrates, like the physiologically-significant secondary metabolite 3,4-Dimethoxybenzyl (Veratryl) alcohol [11], can originate, firstly, through *de novo* biosynthesis [12] and secondly, through reduction of aromatic aldehydes released during lignin degradation in cyclic redox reactions involving also aryl-alcohol dehydrogenase (Aadp) [13-17]. Furthermore, the identification of the Veratryl alcohol binding site in LiP [18] confirms the presumption that this alcohol also serves as a redox mediator for LiP-catalyzed depolymerization of lignin [11].

By following the reduction of 3,4-Dimethoxybenzaldehyde (Veratraldehyde) in Nitrogen-limited cultures of *P. chrysosporium*, Muheim *et al.*[19] purified an intracellular aryl-alcohol dehydrogenase (EC 1.1.1.91) from this lignin-degrading fungus. A cDNA coding for this protein was later isolated and characterized [20]. However, the biochemical properties of the Aadp enzyme were not extensively studied.

Due to its high efficiency in lignin degradation, and to its potential applications in the textile, fuel and paper industries, the 35-Mb haploid genome of *P. chrysosporium* strain RP78 has been sequenced [2]. The current draft release, version 2.0, includes a total of 10,048 gene models [21] and reveals that the secreted oxidases, peroxidases and hydrolytic enzymes that cooperate in wood decay exist as large multi-gene families. Taking advantage of this genome sequence, this work describes the cloning of an *AAD* cDNA and the comprehensive biochemical characterization of the encoded enzyme in order to get deeper insight into its biological relevance and biotechnological applications potential such as the degradation of aromatic inhibitors in lignocellulosic hydrolysates that strongly impair ethanol fermentation by yeast [22], as well as for the microbial production of natural flavour and fragrance molecules like 2-Phenylethanol.

## Results and discussion

### Cloning of a cDNA from *Phanerochaete chrysosporium* encoding an aryl-alcohol dehydrogenase

Using the amino acid sequence coded by a previously cloned *AAD* ORF from *Phanerochaete chrysosporium* (*Pc*) strain OGC101 [20] as query, a BLAST alignment was performed against the translated predicted ORFs of the genome sequence of *P. chrysosporium* strain RP78 [2,21]. The results showed the existence of 8 *AAD* homologues that consist of six to nine exons and encode proteins from 240 to 398 amino acids. The presence of multiple *AAD* genes in the *Pc* genome is in accordance with strong multiple bands observed in a Southern blot by Reiser *et al.*[20]. Interestingly, in scaffold_1, two tandem *AAD* homologues (scaffold_1:1025231 to 1023962, and scaffold_1:1027063 to 1025827) were found adjacent to each other. The distance between these two adjacent ORFs is only 596 base-pairs. This extensive genetic diversity was also observed for other lignin-biodegradation related genes encoding peroxidases, oxidases, glycosydases and cytochrome P450s [2]. The existence of multiple *AAD* genes might suggest multiple specificities required to reduce various aryl-aldehydes arising from the catabolism of complex wood polymers. 

**Figure 1:**
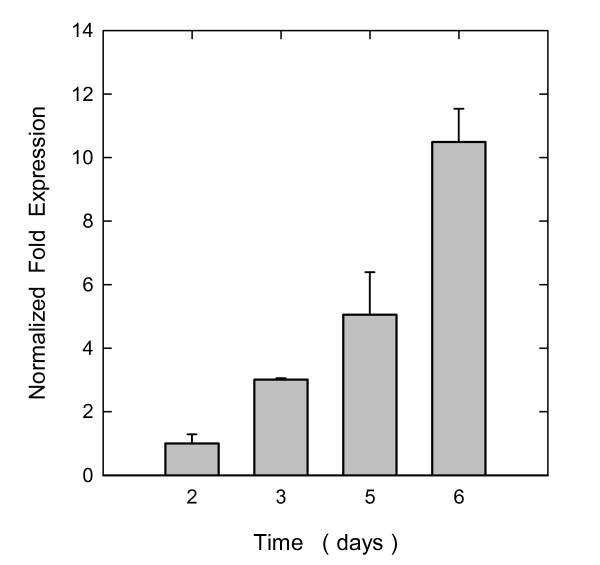
**Expression of *Pc AAD1 *gene during Nitrogen-limited cultivation.** The *Pc AAD1* transcript level was evaluated by real-time PCR with β-Tubulin as reference gene. Day 2 sample was taken as the calibrator sample. Results are the mean ± SEM from technical triplicates of four biological replicates.

Among the 8 predicted homologous ORFs in the genome of *Pc* strain RP78, the one in scaffold_3:2235704–2237287 (JGI Transcript Id: 11055) has only 37 base pairs differences with the cDNA previously cloned by Reiser *et al.*[20] and encodes a 100% identical amino acid sequence. Considering that the remaining 7 *AAD* homologues show 72.1, 66.7, 64.6, 55, 54.1, 49.9 and 45.7% amino acid identity with this cDNA sequence, we designed specific primers on the coding region from scaffold_3:2235704–2237287 (hereafter termed *AAD1*) to clone the full length cDNA using RACE (rapid amplification of cDNA ends, [23,24]) and PCR techniques. The method was adopted because of the presence of 5 introns in the genomic sequence of this *Pc AAD1* gene. The RNA used for this cloning was obtained from a six days Nitrogen-limited culture of *Pc* strain BKM-F-1767. As shown in Figure 1, qPCR assays under this growth condition showed that the *AAD1* transcript began to accumulate at day 2 and continued over 6 days. This result nicely correlated with an increase of aryl-alcohol dehydrogenase activity acting on Veratraldehyde during N-limited culture and reaching a maximum after 6 days of growth [19]. The RACE-PCR method on the 6-days purified RNA allowed us to isolate a 1.4 kilobase full-length cDNA containing a 1155 bp ORF that encoded a protein 100% identical with the translated genomic sequence from *Pc* RP78 strain [2,21] as well as with that of Reiser *et al.*[20]. The sequencing results of the cloned *Pc AAD1* cDNA also showed the presence of a 5′ untranslated region (UTR) and of a 3′ poly(A) tail, confirming the integrity of the mRNA template. Comparison of the 5′UTR (159 nucleotides in total) with that of the cDNA by Reiser *et al.*[20] revealed 94.3% nucleotide identity, suggesting they are the same gene in the two strains.

### Heterologous expression in *E. Coli* and purification of recombinant *Pc* Aad1p

**Figure 2:**
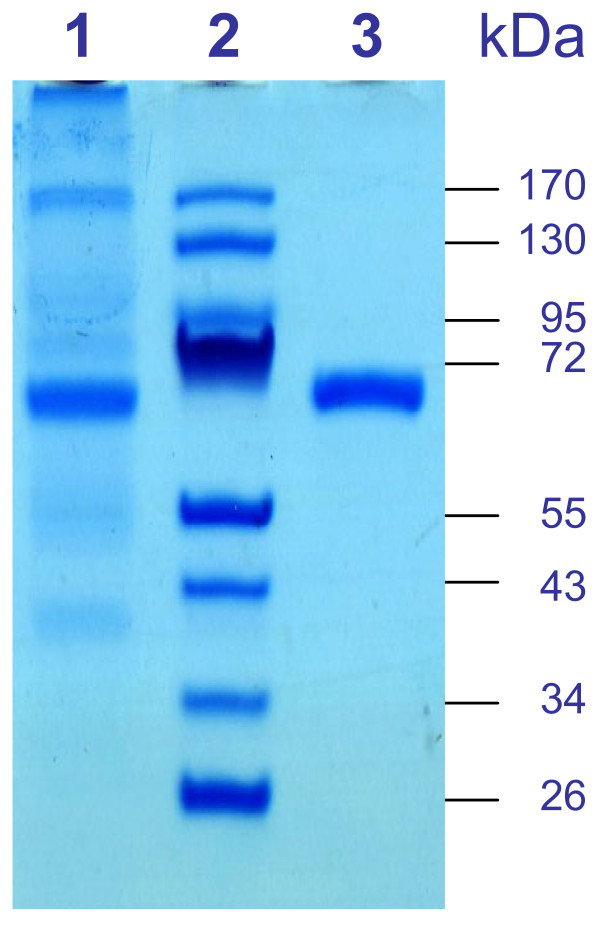
**Purification of the recombinant *Pc *Aad1p after expression in *E. coli.*** The *Pc* Aad1p fused to GST and His_6_ tags was expressed in *E. coli* BL21 Star™(DE3) strain with the pGS-21a expression vector under the control of the strong T7 promoter. Proteins were separated by SDS-PAGE and visualized by Coomassie Blue staining. *Lane 1*: Cell lysate of *E. coli* IPTG-induced cells; *Lane 2*: Protein molecular size markers; *Lane 3*: Recombinant *Pc* Aad1p after purification by Glutathione-affinity chromatography.

In order to obtain large amounts of purified recombinant enzyme for biochemical characterization, the *Pc AAD1* ORF was cloned in pGS-21a and pGEX-6p-1 vectors and expressed in *E. coli* to produce GST and/or His_6_ tagged proteins. The expression conditions were optimized using different *E. coli* strains, cultivation temperatures, IPTG concentrations and induction times. The highest accumulation of recombinant *Pc* Aad1p was obtained with *E. coli* BL21 Star™(DE3) strain harbouring the pGS-21a-*AAD1* expression vector after overnight induction with 0.1 mM IPTG at 16°C allowing the production of up to 1.8 ± 0.1 g·L^−1^ of recombinant protein after purification. After cell disruption, the recombinant Aad1p was purified by Glutathione affinity chromatography to yield a single protein band as shown on SDS-Polyacrylamide gel electrophoresis (Figure 2, lane 3). This SDS-PAGE also showed that the recombinant protein was the major band in the cell lysate (Figure 2, lane 1) and that the purified protein migrates at an apparent molecular mass of 70 kDa in our conditions of electrophoresis. Taking into account the presence of the GST and His_6_ tags in the fusion protein, which correspond to ~ 30 kDa, the molecular mass of our purified *Pc* Aad1p is in accordance with the theoretical molecular mass calculated from its amino acid composition (43 kDa) and very close to the apparent 47 kDa of the Aad enzyme purified from *P. chrysosporium* by Muheim *et al.*[19].

### Biochemical characterization of the purified recombinant *Pc* Aad1p

#### Structure analysis of Pc Aad1p

We searched for functional domains of the *Pc* Aad1 protein using the Pfam database server [25,26]. This *in silico* analysis identified the protein as belonging to subfamily AKR9A of the aldo-keto reductase (AKR) superfamily with residues D71, Y76 and K103 as predicted active- sites. The AKR superfamily is one of the three enzyme superfamilies that perform oxidoreduction on a wide variety of natural and foreign substrates [27]. The large AKR superfamily includes presently 15 families, with more than 170 proteins identified in mammals, plants, fungi and bacteria. AKR structures share a highly conserved (α/β)_8_-barrel motif, a conserved cofactor (mostly NADPH) binding site and catalytic tetrad, and a variable loop structure which usually defines broad substrate specificity. The majority of AKRs are monomeric proteins of about 320 amino acids in length, although several members from families AKR2, AKR6 and AKR7 were found to form multimers [28]. The closest AKR protein ‘relatives’ of *Pc* Aad1p (AKR9A3) are the fungal norsolorinic acid reductase from *Aspergillus flavus* (AKR9A2) and sterogmatocystin dehydrogenase from *Aspergillus nidulans* (AKR9A1) and the putative yeast proteins Aad14p, Aad3p, Aad4p and Aad10p from *Saccharomyces cerevisiae*. According to the family tree structure, the nearest AKR with 3D structure characterized is AKR11C1 from the bacterium *Bacillus halodurans*[27,29]. Aldo-keto reductases catalyze oxidation and reduction reactions on a range of substrates using NAD(P)(H) as cofactor. An ordered Bi Bi kinetic mechanism, in which cofactor binds first and leaves last, has been demonstrated for pig kidney aldehyde reductase (ALR) [30], bovine kidney aldose reductase ADR [31], rat liver 3-alpha-hydroxysteroid dehydrogenase (3α-HSD) [32] and 3-oxo-5b-steroid 4-dehydrogenase [33], and may be a characteristic feature of other AKRs [34]. The reduction reaction involves 4-pro-R hydride transfer from NAD(P)H to the substrate carbonyl and protonation of the Oxygen by a residue of the enzyme acting as a general acid [34]. The rate of this reaction is increased with substrates harbouring chemical structures that facilitate their nucleophilic attack by the hydride ion. It is also influenced by the orientation and/or relative mobility of the carbonyl function with respect of the rest of the molecule that would affect its protonation by one or more possibly acid residues of the active site.

#### Temperature- and pH-dependence of Pc Aad1p activity

**Figure 3:**
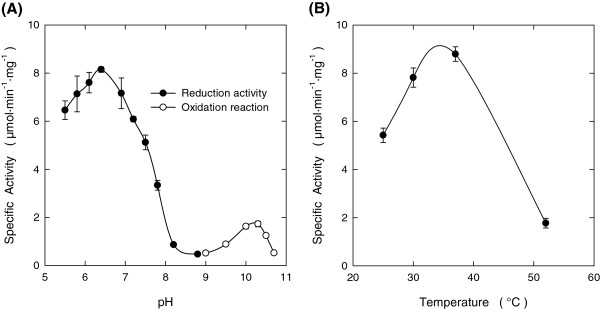
**pH and temperature dependence of recombinant *Pc *Aad1p activity.(A)** Effect of pH in reduction and oxidation reactions. Reduction activities were measured at pH 5.5-8.8 with 0.2 mM NADPH and 0.2 mM 3,4-Dimethoxybenzaldehyde. Oxidation reactions were performed at pH 9.0-10.7 using 0.3 mM NADP ^+^ and 10 mM 3,4-Dimethoxybenzyl alcohol. Reactions were carried out at 30°C. **(B)** Effect of temperature on the reduction activity of recombinant *Pc* Aad1p. Activity was measured at pH 6.1 with NADPH and 3,4-Dimethoxybenzaldehyde at 0.2 mM final concentration. The reaction was started by adding 9.0 μg of the enzyme. Results are the mean ± SEM from two separate experiments.

To determine pH and temperature optimum of the recombinant purified *Pc* Aad1p, we used Veratraldehyde as substrate for the reductive sense, and the corresponding alcohol for the oxidative sense of the reaction, while NADP(H) was used as the cofactor. As shown in Figure 3A, the activity of this enzyme was optimal at about pH 6.4 in the reductive sense whereas oxidation rates could only be measured in basic conditions with an optimum at pH 10.4. At this pH, the oxidation activity was 7-fold lower than at the optimal pH for the reductive reaction. These results strongly support the fact that the *Pc* Aad1p works in the cells predominantly as an aldehyde reductase. The optimal temperature for activity was only determined in the reductive sense and was found to be close to 37°C (Figure 3B).

#### Substrate specificity and kinetic properties of Pc Aad1p

**Figure 4:**
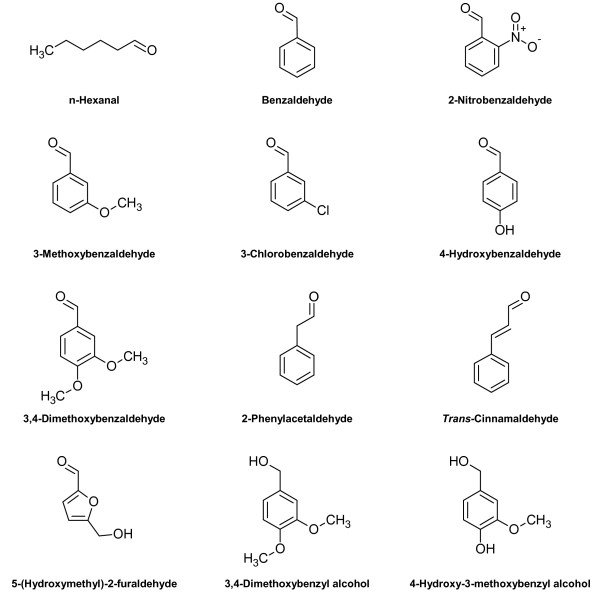
**Chemical structures of several substrates of recombinant *Pc *Aad1p.** Chemical structure of some of the aldehyde and alcohol substrates of *Pc* Aad1p analyzed in this study ordered by chemical function and substitution: aliphatic aldehydes (n-Hexanal), aryl-aldehydes (Benzaldehyde and related compounds, 2-Phenylacetaldehyde and *trans*-Cinnamaldehyde) and aryl-alcohols. Other substrates are presented in Table 1 and 2.

The substrate specificity of the purified recombinant *Pc* Aad1p protein was determined with a large spectrum of chemical molecules including linear aliphatic and aryl-aldehydes and alcohols, and ethyl-, ramified and aryl acetate esters (Table 1), keeping in mind that the presence of a GST tag at the amino terminus could modify the enzyme properties. Figure 4 shows some of the aldehyde and alcohol substrates analyzed in this study ordered by chemical function and substitution. For comparative analysis, we carried out our assays at pH 6.1 in 50 mM MES and at 30°C using the same concentration of substrate molecules and NADPH and compared the measured activity to that obtained with Veratraldehyde, which was used as the reference. The activity value with this substrate was set to 100%. As indicated in Table 1, *Pc* Aad1p activity with mono-methoxylated Benzaldehyde at positions 3 (meta) or 4 (para), or dimethoxylated at positions 3,5 was very close or even slightly higher than with Veratraldehyde (3,4-Dimethoxybenzaldehyde). Activity was reduced by two when the methoxy radical was on carbon 2 (ortho). The presence of a hydroxyl group on Benzaldehyde or on methoxy-substituted Benzaldehyde resulted in a dramatic drop of the activity of *Pc* Aad1p. Likewise, the enzyme was 3 to 5-fold less active on other types of substitutions of the Benzaldehyde molecule such as with Chlorine, Fluorine or Nitro functional groups. Furthermore, the *Pc* Aad1p activity on Phenylacetaldehyde was comparable to that of Veratraldehyde. Linear aldehydes of 3 to 11 carbon atoms were also assayed for substrate specificity of *Pc* Aad1p. The highest activity was observed on C6 to C8 aldehydes, with reaction rates about 2-fold lower than on Veratraldehyde but comparable to that on Benzaldehyde. No activity was detected for Propanal (C3) and Butanal (C4) and very low activity for C9 to C11 aldehydes.

**Table 1:** **Substrate specificity of the recombinant Aad1p from *Phanerochaete chrysosporium***

**Reduction**	**Activity (%)**	**Oxidation**	**Activity(%)**
*Linear aliphatic aldehydes*		*Aryl-alcohols*	
Propanal (C3)	nd	4-Methoxybenzyl alcohol	15
Butanal (C4)	nd	3,4-Dimethoxybenzyl alcohol	100
Pentanal (C5)	8	3,5-Dimethoxybenzyl alcohol	3
Hexanal (C6)	47	3,4,5-Trimethoxybenzyl alcohol	5
Heptaldehyde (C7)	26	4-Hydroxybenzyl alcohol	3
Octanal (C8)	37	3-Hydroxy-4-methoxybenzyl alcohol	8
Nonanal(C9)	3	4-Hydroxy-3-methoxybenzyl alcohol	46
Decanal (C10)	2		
Undecanal (C11)	1		
*Aryl-aldehydes*		**No detectable activity**	
Benzladehyde	42	*Linear aliphatic alcohols*	
2-Methoxybenzaldehyde	58	Ethanol (C2)	
3-Methoxybenzaldehyde	117	Propanol (C3)	
4-Methoxybenzaldehyde	110	Butanol (C4)	
3,4-Dimethoxybenzaldehyde	100	Pentanol (C5)	
3,5-Dimethoxybenzaldehyde	110	Hexanol (C6)	
4-Hydroxybenzaldehyde	34	Octanol (C8)	
3-Hydroxy-4-methoxybenzaldehyde	17	Nonanol (C9)	
4-Hydroxy-3-methoxybenzaldehyde	19	Decanol (C10)	
3-Chlorobenzaldehyde	73	Undecanol (C11)	
4-Chlorobenzaldehyde	61	*Ramified aliphatic alcohols*	
2-Nitrobenzaldehyde	57	2-Methylpropanol (C4)	
3-Nitrobenzaldehyde	25	2-Methylbutanol (C5)	
4-Nitrobenzaldehyde	31	*Aryl-alcohols*	
2-Fluorobenzaldehyde	27	Benzyl alcohol	
3-Fluorobenzaldehyde	69	2-Methylbenzyl alcohol	
4-Fluorobenzaldehyde	29	3-Methylbenzyl alcohol	
Phenylacetaldehyde	109	4-Methylbenzyl alcohol	
*trans*-Cinnamaldehyde	39	2-Methoxybenzyl alcohol	
*Others*		3-Methoxybenzyl alcohol	
5-(Hydroxymethyl)-2-furaldehyde	36	2-Chlorobenzyl alcohol	
		4-Chlorobenzyl alcohol	
**No detectable activity**		2-Nitrobenzyl alcohol	
*Aryl-acid*		3-Nitrobenzyl alcohol	
Phenylacetic acid		4-Nitrobenzyl alcohol	
*Ethyl-esters*		2-Fluorobenzyl alcohol	
Ethyl hexanoate		3-Fluorobenzyl alcohol	
Ethyl octanoate		4-Fluorobenzyl alcohol	
Ethyl decanoate		2-Phenylethanol	
*Ramified and aryl acetate esters*		*trans*-Cinnamyl alcohol	
2-Methylpropyl acetate		*Aryl-acid*	
2-Methylbutyl acetate		Phenylacetic acid	
3-Methylbutyl acetate		*Ethyl-esters*	
2-Phenylethyl acetate		Ethyl hexanoate	
*Others*		Ethyl octanoate	
L-Glutathione oxidized		Ethyl decanoate	
		*Ramified and aryl acetate esters*	
		2-Methylpropyl acetate	
		2-Methylbutyl acetate	
		3-Methylbutyl acetate	
		2-Phenylethyl acetate	

Results are the mean of three separate experiments with relative SEM being lower than 6%.

Among the substrates assayed for the oxidation reaction by *Pc* Aad1p with NADP^+^ as cofactor, the highest activity was by far that on Veratryl alcohol (3,4-Dimethoxybenzyl alcohol), whereas other mono-, di- or tri-substituted methoxybenzyl alcohols showed poor reactivity with this enzyme. Interestingly, the *Pc* Aad1p showed 46% activity on 4-Hydroxy-3-Methoxybenzyl alcohol (Vanillyl alcohol) as compared to that on Veratryl alcohol. No activity could be detected on many other linear aliphatic, ramified aliphatic or aryl alcohol substrates as well as on some acetate esterified aryl and ramified alcohols. Altogether, these results suggest that a specific size, structure and conformation of the substrate are necessary to allow concurrent interactions of the carbonyl group of the substrate molecule with the cofactor and with key amino acids of the active site. Other parameters like the relative hydrophilic/hydrophobic character of the substrates and of the active site as well as the possibility of resonance delocalization within a conjugated π system of the substrate might also account for relative specificity of the Aad1p enzyme to its substrate.

**Figure 5:**
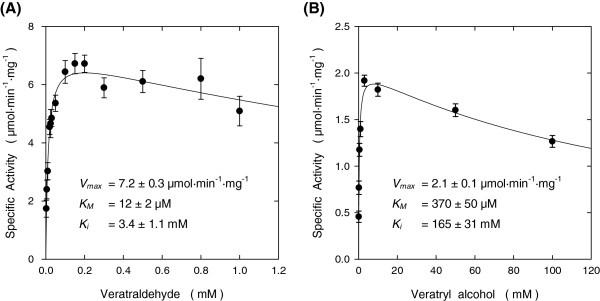
**Kinetic parameters of recombinant *Pc *Aad1p for Veratraldehyde and Veratryl alcohol.** The kinetic parameters of the *Pc* Aad1 enzyme were determined for **(A)** the reduction reaction of Veratraldehyde and **(B)** the oxidation reaction of Veratryl alcohol. Activities were measured at 30°C in 50 mM MES buffer at pH 6.1 containing 0.3 mM NADPH in the reduction sense and in 100 mM Glycine-KOH buffer at pH 10.3 with 0.3 mM NADP^+^ for the oxidation reactions. The kinetic parameters for other substrates are presented in Table 2. Results are the mean ± SEM from at least three separate experiments.

We then obtained precise kinetic parameters of *Pc* Aad1p with respect to cofactor dependency and affinity to several substrates like Veratraldehyde or Veratryl alcohol (Table 2). In the reductive sense, using 0.2 mM Veratraldehyde, the activity of *Pc* Aad1p for NADPH oxidation followed a Michaelis-Menten curve with an apparent *K*_*M*_ = 39 μM. NADH could also be used as electron donor though exhibiting a lower affinity (*K*_*M*_ = 220 μM). The enzyme was only active with NADP^+^ in the oxidation sense of the reaction, with a *K*_*M*_ of 38 μM. Moreover, the activity of this enzyme determined against Veratraldehyde or Veratryl alcohol using NADPH or NADP^+^ as cofactor showed a slight inhibition at elevated concentration of substrate (Figure 5). However, the apparent *K*_*M*_ for Veratraldehyde was 30-fold that for Veratryl alcohol. This explained also that the catalytic efficiency *k*_*cat*_*/K*_*M*_ of *Pc* Aad1p was about 100-fold higher in the reductive than in oxidative sense of the reaction. Reduction activity towards Veratraldehyde has also been described for the enzymes Adh6p and Adh7p from the yeast *Saccharomyces cerevisiae*[35-37].

**Table 2:** **Kinetic parameters of the recombinant Aad1p from*Phanerochaete chrysosporium***

	**K**_***M***_**μM**	**K**_***cat***_**min**^**-1**^	**k**_***cat***_**/K**_***M***_**μM**^**-1**^**·min**^**-1**^	**K**_***i***_**μM**
**Substrates**				
* Reduction*				
3,4-Dimethoxybenzaldehyde	12 ± 2	530 ± 25	44 ± 9	3400 ± 1100
3,5-Dimethoxybenzaldehyde	22 ± 4	590 ± 30	27 ± 6	2100 ± 600
4-Methoxybenzaldehyde	90 ± 10	490 ± 10	5.4 ± 0.7	ni
5-(Hydroxymethyl)-2-furaldehyde	270 ± 40	176 ± 6	0.65 ± 0.12	136000 ± 28000
Phenylacetaldehyde	530 ± 90	670 ± 25	1.3 ± 0.3	ni
3-Hydroxy-4-methoxybenzaldehyde	1400 ± 900	230 ± 110	0.16 ± 0.18	2300 ± 1800
4-Hydroxy-3-methoxybenzaldehyde	1400 ± 600	200 ± 50	0.14 ± 0.10	5100 ± 2300
Benzaldehyde	1700 ± 600	430 ± 50	0.3 ± 0.1	81000 ± 44000
* trans*-Cinnamaldehyde	3400 ± 1300	670 ± 200	0.2 ± 0.1	3500 ± 1600
* Oxidation*				
3,4-Dimethoxybenzyl alcohol	370 ± 50	153 ± 6	0.41 ± 0.07	165000 ± 31000
4-Hydroxy-3-methoxybenzyl alcohol	25000 ± 7000	260 ± 60	0.010 ± 0.005	ni
**Coenzymes**				
* Oxidation*				
NADPH	39 ± 5	680 ± 30	17 ± 3	ni
NADH	220 ± 130	120 ± 40	0.6 ± 0.5	ni
* Reduction*				
NADP^+^	38 ± 7	154 ± 7	4.1 ± 0.9	ni
NAD^+^	nd	nd	nd	nd

*nd:* no detectable activity under the conditions of the assay.

*ni:* no inhibition detected.

## Conclusion

This study describes the cloning and biochemical properties of an aryl-alcohol dehydrogenase of the white-rot fungus *Phanerochaete chrysosporium*. It also shows its wide spectrum of activity on various chemicals (natural and non-natural) such as linear aliphatic and aryl-aldehydes, as well as its preference to function in the reductive sense under physiological conditions. This enzyme can be considered in the design of metabolic engineering strategies/synthetic biology systems for biotechnological applications such as the degradation of aromatic inhibitors present in lignocellulosic hydrolysates that impair yeast fermentation, or the microbial production of natural flavours and fragrances like the rose-like flavour compound 2-Phenylethanol. Further studies on the crystal structure of the protein and the determination of the key amino acids in its active site would be extremely helpful for implementing protein engineering strategies in order to modify or improve the kinetic parameters of the enzyme.

## Materials and methods

### Materials

DNA oligonucleotides were synthesized by Eurogentec (Seraing, Belgium). Phusion® High fidelity DNA polymerase, Taq DNA polymerase, restriction enzymes and T4 DNA ligase were from New England Biolabs (Ozyme, Saint-Quentin-en-Yvelines, France). dNTPs were from Eurogentec (Seraing, Belgium). Plasmids were sequenced by Beckman Coulter Genomics (Grenoble, France). Bacterial and fungus culture media were from Difco (Detroit, MI, USA). Glutathione Sepharose™ 4B was from GE Healthcare Bio-Sciences AB (Uppsala, Sweden). Lysozyme and reduced and oxidized L-Glutathione were from Sigma-Aldrich Chimie SARL (Saint-Quentin Fallavier, France). SDS-PAGE gels were made with proteomics grade NEXT GEL 12.5% acrylamide solution from AMRESCO (Solon, OH, USA). PageBlue™ protein staining solution and PageRuler™ (cat. #SM0671) protein molecular size markers were from Fermentas (Thermo Electron SAS, Villebon sur Yvette, France). QIAquick Gel Extraction Kit was employed for purifying PCR products from gels. Plasmid extraction was done with QIAprep Spin Miniprep kit (Qiagen SAS, Courtaboeuf, France). Chemical substrates were purchased at highest available purity from Sigma-Aldrich Chimie SARL (Saint-Quentin-Fallavier, France). Unless otherwise specified, all other products were from Sigma-Aldrich Chimie SARL. Protein concentration was determined with the Bio-Rad Protein Assay (Bio-Rad, Marnes-la-Coquette, France) based on the Bradford method [38] using bovine serum albumin as calibration standard. Crude and purified protein extracts were analyzed by SDS-PAGE and visualised by Coomassie blue staining.

### Strain and growth conditions

The white-rot basidiomycete *Phanerochaete chrysosporium* BKM-F-1767 strain used in this study (CBS 481.73) was purchased from Centraalbureau voor Schimmelcultures (Utrecht, Netherlands) in the form of a freeze-dried fungal culture. The mycelium was inoculated on freshly prepared Difco™ Potato Dextrose Agar (PDA) plates and incubated at 37°C for four days before storage and maintenance at 4°C on PDA plates or at −80°C in 30% glycerol for long-term preservation. Spore suspensions were prepared after 4-days propagation at 37°C on PDA plates by washing the agar surface with 10 mL of 50 mM sodium acetate buffer at pH 4.5. Spore counts were determined with a counting chamber Thoma double cell.

To induce *AAD1* expression in *P. chrysosporium*, 600 mL of Nitrogen-limited liquid medium was inoculated at 10^4^ spores.mL^-1^ in a 1 L Erlenmeyer flask and cultivated at 37°C and 150 rpm on a TR-225 rotary shaker (Infors AG, Bottmingen, Switzerland) for 1 week. The medium was composed of basal elements, trace elements and vitamins according to [39-41]: (a) *Basal elements*: Glucose 56 mM, Ammonium tartrate 1.19 mM, KH_2_PO_4_ 7.35 mM, MgSO_4_·7H_2_0 2.02 mM, CaC1_2_·2H_2_0 0.68 mM, FeSO_4_·7H_2_0 6.47 × 10^−2^ mM, Nitrilotriacetate 7.85 μM; (b) *Trace elements*: MnSO_4_·H_2_0 5.92 μM, CoC1_2_·6H_2_0 4.20 μM, ZnSO_4_·7H_2_0 10.4 μM, CuSO_4_·5H_2_0 0.04 μM, AlK(SO_4_)_2_ 2.28 × 10^−2^ μM, H_3_BO_3_ 0.162 μM, Na_2_MoO_4_ 4.86 × 10^−2^ μM; (c) *Vitamins*: Biotin 8.19 nM, Folic acid 4.53 nM, Thiamine hydrochloride (B1) 0.148 μM, Riboflavin 0.133 μ M, Pyridoxine hydrochloride (B6) 48.6 μM, Cyanocobalamin (B12) 7.38 × 10^−2^ nM, Nicotinic acid 40.6 nM, D-Calcium pantothenate 20.9 nM, p-Aminobenzoic acid 36.5 nM, Thioctic acid 24.2 nM. The pH of the basal elements solution was adjusted to 4.5 with 20% (m/v) NaOH. Trace elements and vitamins were prepared in 10000-fold concentrated stock solutions and added to the basal solution after autoclaving at 120°C for 20 min.

### Analysis by qPCR of *Phanerochaete chrysosporium AAD1* gene expression

The expression of *Pc AAD1* during Nitrogen-limited cultivation was analyzed by real-time PCR (qPCR). The frozen mycelia were disrupted with TissueLyser II grinder for 2 x 1.5 min at 30 s^−1^ frequency (Qiagen SAS, Courtaboeuf, France) and total RNA was purified from c.a. 100 mg wet-mycelium with the RNeasy Plant Mini Kit (Qiagen) according to the manufacturer’s instructions. The quality of the extracted RNA was determined using the Bioanalyzer 2100 with the RNA 6000 Nano LabChip kit (Agilent Technologies, Massy, France) and quantified in the NanoDrop ND-1000 UV-visible light spectrophotometer (Fisher Scientific SAS, Illkirch, France). cDNA was then synthesized from an exact amount of 1 μg total RNA in 20 μL reaction mixtures using the iScript™ cDNA Synthesis Kit (Bio-Rad, Marnes-la-Coquette, France). Real-time PCR reactions were carried out using a MyiQ Single-Color Real-Time PCR Detection System (Bio-Rad). The β-Tubulin transcript coded by scaffold_10:459524–461702 was amplified in parallel with the target *AAD1* cDNA and used as reference for normalization of gene expression. The stable Ct values observed for this gene among the different samples reflects the stability of its expression under the conditions tested. Primer sequences were as follows: AAD1-2-3-F2 (5′-TCGTTGCTACCAAGTACAGTCTGGTCTACAAACGGGG-3′) and AAD1-3-4-R2 (5′-GCGATGGCCATCCCTTCGTGAATGCACA-3′) for target gene *Pc AAD1*;x BTUB-N-Term-F (5′-ATCGGTGCCAAGTTCTGGGAGGT-3′) and BTUB-N-Term-R (5′-TGTTCGCGCCAACTTCGTTGTAGT-3′) for reference gene. Reactions were performed in 25 μL final reaction volume using iQ™ SYBR® Green Supermix (Bio-Rad), 0.1 μM final concentration of each primer and 1 μL of the cDNA preparation. The qPCR conditions were as follows: 1 cycle (95°C for 3 min), 40 cycles (95°C for 16 s, and 58°C for 30 s). Reactions were set up in triplicate for each of four biological replicates to ensure the reliability of the results. The absence of genomic DNA in RNA samples was checked by real-time PCR before cDNA synthesis. Melting curves (55-95°C, in 0.5°C increments for 30 s) were performed at the end of the qPCR reaction to verify the specificity of the amplification products and the absence of primer dimers.

### RACE cloning of *AAD1* cDNA from *Phanerochaete chrysosporium*

The relative expression level of *AAD1* gene in *P. chrysosporium* being maximum after six days of cultivation in nitrogen-limited liquid medium, fungus pellets were harvested at this physiological state, filtered, washed twice with water and frozen in liquid nitrogen. The frozen mycelia were disrupted 2 x 1.5 min at 30 s^-1^ frequency with TissueLyser II grinder (Qiagen SAS, Courtaboeuf, France) and total RNA was purified from c.a. 100 mg wet-mycelium with the RNeasy Plant Mini Kit (Qiagen). In order to clone the *P. chrysosporium AAD1* full-length cDNA, 5′- rapid amplification of cDNA ends (RACE) and 3′-RACE were performed with the SMART™ RACE cDNA amplification kit from Clontech (Ozyme, Saint-Quentin-en-Yvelines, France). After separate synthesis by reverse transcription, 5′- and 3′-RACE cDNA fragments were amplified by touchdown PCR in independent reactions with the gene specific primers AAD1-3-4-R2 (5′GCGATGGCCATCCCTTCGTGAATGCACA-3′) and AAD1-2-3-F2 (5′-TCGTTGCTACCAAGTACAGTCTGGTCTACAAACGGGG-3′), respectively. Touchdown PCR conditions were as follows: 5 cycles (94°C for 30 s, 72°C for 3 min), 5 cycles (94°C for 30 s, 70°C for 30 s and 72°C for 3 min); then 25 cycles (94°C for 30 s, 68°C for 30 s, and 72°C for 3 min). The resulting amplicons were cloned into pGEM®-T Easy vector (Promega, Charbonnieres, France). The full-length *Pc AAD1* ORF was obtained by overlapping PCR using Phusion® High-Fidelity DNA Polymerase (Ozyme, Saint-Quentin-en-Yvelines, France), the 5′- and 3′RACE cloned fragments as templates and the AAD1-ORF-Start-F (5′-ATGAACATCTGGGCACCCGCA-3′) and AAD1-ORF-End-R (5′CTACTTCTGGGGGCGGATAGC-3′) primers. Thermal cycling conditions were: 1 cycle at 95°C for 4 min, followed by 25 cycles of 95°C for 30 s, 68°C for 30 s and 72°C for 3 min. The resulting PCR product was cloned into the pGEM®-T Easy vector (Promega). All PCR products were A-tailed before cloning into pGEM®-T Easy vector and transferring into chemically competent *E. coli* DH5Î± cells (Invitrogen™, Life Technologies SAS, Saint Aubin, France). The inserts were sequenced at Beckman Coulter Genomics (Grenoble, France).

### Expression and purification of *Pc AAD1* ORF in *Escherichia coli*

The full-length *Pc AAD1* ORF obtained by RACE cloning was amplified by Phusion® DNA polymerase PCR with primers BamHI-Start-F (5′-CCTGGGATCCATGAACATCTGGGCACCCGCA-3′) and NotI-NoStop-R(5′-GAGCGGCCGCCTTCTGGGGGCGGATAGCCTG-3′) in order to generate *Bam*HI and *Not*I sites (underlined in the sequence) respectively at 5′ and 3′ of the *AAD1* ORF and cloned in pGEM®-T Easy vector (Promega). PCR conditions were: 1 cycle (98°C for 30 s), 30 cycles (98°C for 10 s, 65°C for 30 s and 72°C for 45 s); then 1 cycle (72°C for 7 min). Insert was excised from vector by digestion with *Bam*HI and *Not*I and directionally subcloned into the expression vector pGS-21a (GenScript) previously digested with the same restriction enzymes. The resulting construct, termed pGS-21a-*AAD1*, was sequenced to verify that the PCR reaction had not introduced any mutations. This expression plasmid encoded the recombinant fusion protein containing a His_6_-GST tag at the N-terminus and a His_6_ tag at the C-terminus.

Three *E. coli* strains BL21 Star™ (DE3) (Invitrogen™, Life Technologies SAS, Saint Aubin, France), BL21(DE3) and BL21- CodonPlus(DE3)-RIL (Stratagene, Agilent Technologies, Massy, France) were tested as expression hosts after transformation with plasmid pGS-21a-*AAD1*. Overnight cultures of the transformants made in LB medium containing the appropriate antibiotic(s) at 37°C were used to inoculate 150 mL of the same medium in 1 L Erlenmeyer flasks at an initial OD_600_ of 0.1. The bacterial biomass was grown at 37°C and 100 rpm until OD_600_ 0.7–0.9. The production of the recombinant protein was induced by addition of Isopropyl β-D-1-thiogalactopyranoside (IPTG) at 0.1 mM final concentration followed by incubation at 16°C and 120 rpm for 12 h. Bacterial cells were collected by centrifugation (4°C, 10000 g, 1 min), resuspended in PBS buffer at pH 7.3 containing 200 μg·mL^-1^ Lysozyme and disrupted by sonication (ten 30 s pulses with a Vibra Cell™ 72434 ultrasonicator operating at 35% power in 25 W scale). After addition of Triton® X-100 at 1% (v/v) final concentration, the cell lysate was left on ice for 20 min and centrifuged (4°C, 10000 g, 20 min) to remove cell debris.

The recombinant *Pc* Aad1p fusion protein was purified by a single-step batch affinity chromatography process on Glutathione Sepharose™ 4B previously equilibrated with PBS buffer at pH 7.3 according to the manufacturer’s instructions. The Glutathione Sepharose™ 4B beads (0.75 mL) were added to the cell lysate supernatant (15 mL) and incubated 2 h at 4°C under gentle agitation (end-over-end rotation) in 50 mL Falcon™ Conical Tubes (BD Biosciences, NJ, USA). Non-adsorbed proteins were removed by washing the beads with PBS buffer at pH 7.3 several times until the Bradford assay for protein did not react any more. The recombinant protein was eluted with 50 mM Tris–HCl, pH 8.0, containing 10 mM reduced L-Glutathione and stored at 4°C.

### Enzyme assays

Enzymatic activity of *Pc* Aad1p was determined spectrophotometrically using an Agilent HP 8453 UV-visible spectrophotometer (Agilent Technologies, Massy, France). Unless otherwise specified, all assays were carried out at 30°C in 1 mL reaction mixtures using 1 cm optical path length microcuvettes. Reactions were initiated by substrate addition and were monitored by recording the absorption at 355 nm. At this wavelength, the molar extinction coefficients of the substrate compounds could be considered as negligible (less than 4%) compared to that of NAD(P)H (ε_355_ = 5.12 mM^-1^.cm^-1^). The effect of pH was studied at 30°C, using 25 mM MES (pH 5.5 − 6.4), 50 mM HEPES (pH 6.9 − 8.2), 25 mM Tris–HCl (pH 8.8) or 100 mM Glycine-KOH (pH 9.0 − 10.7) as buffers. The temperature dependence was evaluated in 50 mM MES buffer (pH 6.1) in the presence of 0.2 mM 3,4-Dimethoxybenzaldehyde and 0.2 mM NADPH and the reaction was started by adding 9.0 μg of the enzyme. The substrate specificity towards a range of substrates (Table 1) and the kinetic parameters determinations (Table 2) were determined in 50 mM MES buffer (pH 6.1) using 0.3 mM NADPH and 1 mM substrate in the reduction sense, or in 100 mM Glycine-KOH buffer (pH 10.3) using 0.3 mM NADP^+^ and 10 mM substrate (except for Octanol where 1 mM was used, and for 2-Chlorobenzyl alcohol and 4-Chlorobenzyl alcohol where 3 mM were used) for the oxidation sense. The specific activity towards 3,4-Dimethoxybenzaldehyde (5.1 μmol·min^-1^·mg^-1^) and to 3,4-Dimethoxybenzyl alcohol (2.0 μmol·min^-1^·mg^-1^) were taken as 100% for the reduction and oxidation reactions, respectively (Table 1).

The kinetic parameters *K*_*M*_, *k*_*cat*_ and *K*_*i*_ for aldehyde and alcohol substrates (Table 2) were computed by fitting initial reaction rates, measured as a function of substrate concentration, to the Michaelis-Menten equation (Equation 1) or, when substrate inhibition was observed, to the uncompetitive substrate inhibition equation (Equation 2) with the non-linear regression Enzyme Kinetics 1.3 module of the SigmaPlot 11.0 package (Systat Software, IL, USA):

(1) $$V=V_{\max}\;\left[S\right]/\left(\;K_{M}+\left[S\right]\;\right)$$

(2) $$V=V_{\max}\;\left[S\right]/\left(K_{M}+\left[S\right]+{\left[S\right]}^{2}/K_{i}\right)\;$$

where *V* represents the reaction rate, *V*_*max*_ is the limiting reaction rate, *S* is the substrate concentration, *K*_*M*_ is the Michaelis constant and *K*_*i*_ is the substrate inhibition constant. The catalytic constant *k*_*cat*_ of the enzyme for the different substrates was derived from $k_{cat\;}=V_{\max}/\left[E\right]$. The total enzyme concentration [E] was evaluated using a protein molecular mass of 74.2 kDa. The enzyme kinetic parameters for NAD(P)H and NAD(P)^+^ + were determined with 0.2 mM 3,4-Dimethoxybenzaldehyde and 10 mM 3,4-Dimethoxybenzyl alcohol, respectively. Results are the mean ± SEM from at least three separate experiments.

## Abbreviations

AAD: Aryl-alcohol dehydrogenase; AAO: Aryl-alcohol oxidase; ADH: Alcohol dehydrogenase; AKR: Aldo-keto reductase; GST: Glutathione S-Transferase; His_6_: Hexahistidine; IPTG: Isopropyl β-D-1-thiogalactopyranoside; LiP: Lignin peroxidise; MnP: Manganese-dependent peroxidise; *Pc*: *Phanerochaete chrysosporium*; RACE: Rapid Amplification of cDNA Ends; PCR: Polymerase Chain Reaction; PDA: Potato Dextrose Agar; qPCR: Real-Time PCR.

## Competing interests

The authors declare that they have no competing interests.

## Authors’ contribution

DDY participated in the design of the study, carried out the experimental work, participated in the interpretation of the results and drafted the manuscript. JMF participated in the design and coordination of this study and helped to revise the manuscript. GMdB conceived and designed the study, coordinated the experiments, interpreted the results and revised the manuscript for important intellectual content. All authors read and approved the final manuscript.
